# Validity of silhouette showcards as a measure of body size and obesity in a population in the African region: A practical research tool for general-purpose surveys

**DOI:** 10.1186/s12963-015-0069-6

**Published:** 2015-12-17

**Authors:** Maryam Yepes, Barathi Viswanathan, Pascal Bovet, Jürgen Maurer

**Affiliations:** University Institute of Social and Preventive Medicine (IUMSP), Lausanne University Hospital, University of Lausanne, Lausanne, Switzerland; Unit for Prevention and Control of Cardiovascular Diseases, Ministry of Health, Victoria, Republic of Seychelles; Department of Economics (DEEP), Faculty of Business and Economics (HEC), University of Lausanne, Lausanne, Switzerland

**Keywords:** Silhouette body size showcard, Obesity, Anthropometric measure, Africa, Social survey, General purpose household surveys, Socioeconomic status

## Abstract

**Background:**

The purpose of this study is to validate the Pulvers silhouette showcard as a measure of weight status in a population in the African region. This tool is particularly beneficial when scarce resources do not allow for direct anthropometric measurements due to limited survey time or lack of measurement technology in face-to-face general-purpose surveys or in mailed, online, or mobile device-based surveys.

**Methods:**

A cross-sectional study was conducted in the Republic of Seychelles with a sample of 1240 adults. We compared self-reported body sizes measured by Pulvers’ silhouette showcards to four measurements of body size and adiposity: body mass index (BMI), body fat percent measured, waist circumference, and waist to height ratio. The accuracy of silhouettes as an obesity indicator was examined using sex-specific receiver operator curve (ROC) analysis and the reliability of this tool to detect socioeconomic gradients in obesity was compared to BMI-based measurements.

**Results:**

Our study supports silhouette body size showcards as a valid and reliable survey tool to measure self-reported body size and adiposity in an African population. The mean correlation coefficients of self-reported silhouettes with measured BMI were 0.80 in men and 0.81 in women (*P* < 0.001). The silhouette showcards also showed high accuracy for detecting obesity as per a BMI ≥ 30 (Area under curve, AUC: 0.91/0.89, SE: 0.01), which was comparable to other measured adiposity indicators: fat percent (AUC: 0.94/0.94, SE: 0.01), waist circumference (AUC: 0.95/0.94, SE: 0.01), and waist to height ratio (AUC: 0.95/0.94, SE: 0.01) amongst men and women, respectively. The use of silhouettes in detecting obesity differences among different socioeconomic groups resulted in similar magnitude, direction, and significance of association between obesity and socioeconomic status as when using measured BMI.

**Conclusions:**

This study highlights the validity and reliability of silhouettes as a survey tool for measuring obesity in a population in the African region. The ease of use and cost-effectiveness of this tool makes it an attractive alternative to measured BMI in the design of non-face-to-face online- or mobile device-based surveys as well as in-person general-purpose surveys of obesity in social sciences, where limited resources do not allow for direct anthropometric measurements.

## Introduction

The rising prevalence of overweight and obesity around the world has sparked substantial interest in the routine measurement of body size and obesity in general-purpose social science surveys. Besides their well-documented role as a risk factor for many adverse health outcomes such as cardiovascular diseases or diabetes [1] or mortality [2], body size and obesity are also commonly associated with important demographic, economic, psychological, and social outcomes such as marital status, productivity and wages, psychological distress, quality of life, and stigma, to name a few [3–10]. Given this wide-ranging role of body size and obesity in determining individual welfare and quality of life, its measurement should not be limited to epidemiological and health studies, but should also become a standard aim of general-purpose social science population surveys.

Obtaining cost-effective yet reliable measures of weight status is, however, often challenging in the context of general-purpose surveys, especially in resource-constrained settings [11]. While measured weight and height remain the gold standard for determining body mass index (BMI) in epidemiological and health studies, many general-purpose social science surveys lack such anthropometric measures due to considerations of survey cost, respondent burden and survey time. Specifically, measured weight and height, as well as complementary measures such as waist and hip circumference, provide reliable assessments of individuals’ body size and obesity; however, they often require a considerable share of (generally limited) survey time, use of specific equipment and know-how for measurement such as weighting scales and stadiometers and trained staff, and impose substantial burden on respondents by requiring them to take off their shoes and (heavy) clothing to obtain accurate measurements [12, 13]. This may be also difficult or impossible to perform in some cultural contexts. Similarly, taking such measurements is generally not feasible in the context of mail and online- or mobile device-based surveys, which require body size measurement based on self-reports. As a result, many general-purpose social science surveys do not contain direct anthropometric measurements of respondents’ weight and height in the face of binding resource constraints and competing survey demands.

A common alternative to anthropometric measurement for assessing individuals’ body size and obesity is to simply ask respondents about their weight and height and compute their BMI based on these self-reports. While self-reports of weight and height are clearly less expensive than measured anthropometrics in terms of survey time, equipment, interviewer training, and respondent burden, their general reliability has often been questioned, especially in studies of older individuals or settings with relatively low levels of literacy [14]. Compared to measured anthropometrics, self-reported weight commonly suffers from significant underreporting, while self-reported height tends to be over-reported, especially among older persons and when comparing men to women [15–19]. Underreporting of weight and over-reporting of height represent mutually reinforcing measurement errors when computing body size as a function of weight for height, which is often further exacerbated through the use of non-linear transformations as in the case of computation of BMI defined as body mass divided by the square of body height. What is more, people with limited literacy often do not know their true weight and height, which can result in significant item non-response and/or low-quality self-reports of individuals’ weight and height [19]. With either one of self-reported weight or height missing, weight for height measures such as BMI cannot be computed at all. The above challenges to obtaining simple and cost-effective, yet reasonably accurate measures of individuals’ body size thus call for new easy-to-use and cost-effective survey instruments for measuring body size, which can be readily employed in general-purpose social science surveys, including in resource-poor settings with potentially low levels of literacy, as well as mail, online or mobile device-based surveys that do not allow for in-person measurements.

**Fig. 1:**
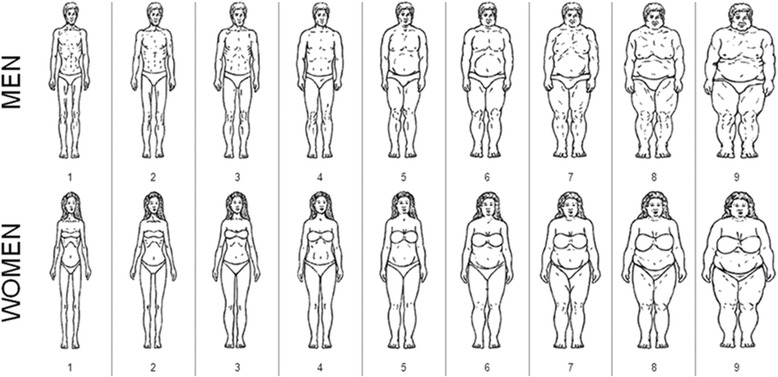
Pulvers’ silhouettes designed for populations of African descent (source: Pulvers 2004, Obesity Res.)

Originally developed by Stunkard and colleagues [20], silhouette showcards, which depict a series of pictures of distinct body sizes, represent an easy visual tool for measuring perceived body size in general-purpose survey settings. Silhouette showcards display sex-specific body sizes, typically in ascending order of BMI (see Fig. 1) [21]. To measure body size, respondents are asked to pick the picture that best represents their own body size. Besides assessing respondents’ own perceived body size, silhouettes have also been used to measure other important concepts in health psychology and nutritional science such as ideal body size for assessing body size dissatisfaction as a potential motivating factor for losing weight through increased exercise or dieting [22–25]. Given the diversity of body sizes by ethnicity, a collection of ethnically-specific silhouette showcards including the original Stunkard silhouettes [20] have been developed and validated as a measure of body size in different populations such as Asians [26, 27], Europeans [28–30], and North and Latin Americans [31–33]. Pulvers and colleagues [21] developed a modified version of these silhouettes based on distinct morphology of African population and used it for body size studies amongst African American population [34–36].

Our study is the first to assess the validity of Pulvers’ silhouettes [21] in an adult population in the African region. Specifically, our study contrasts the self-reported body size obtained from silhouette showcards with comprehensive data from clinical measurements from the Seychelles. This approach allows us to estimate the correlation and the accuracy of showcard-based self-assessments of body-size with a series of clinical indictors of body size and obesity. Our data thus validate the use of silhouette showcards for self-reported body size as a simple, low cost survey instrument for the measurement of body size and obesity in African adults. As a result, our evidence can inform the design of future survey-based assessments of body size and obesity for general-purpose social science surveys, resource-constrained settings or mailed, online, or mobile device-based surveys.

Given their relative simplicity in administration without the need for specialized equipment, training, or respondent knowledge of their weight and height, as well as their low cost in terms of survey time, silhouette showcards could provide a promising tool for cost-effective, reliable measurement of respondents’ weight status in general-purpose social science surveys, in which obtaining measured anthropometric data on height and weight data may not be cost-effective or feasible. Similarly, silhouette showcards can be easily integrated into mailed, online, or mobile device-based surveys for which anthropometric measurement is generally infeasible.

## Methods

### Subjects

We used data from the Seychelles Heart Study IV, a population-based survey conducted in 2013 in the Republic of Seychelles, a group of islands in the Indian Ocean east of Kenya. The Seychelles is a middle-income country and the majority of its population is of African descent. The survey followed the STEPwise approach of the World Health Organisation (WHO) and was approved by the Ministry of Health of the Seychelles following a technical and ethical review [13, 37]. The study sample consisted of 1240 persons aged 25–64 years, randomly selected using sex- and age-stratified sampling based on computerized data of 2010 national population census. Eligible participants were invited by letter to attend the survey and questionnaire and measurements were made by trained survey officers in one main study center in each island. The participation rate of the study was 73 %.

### Survey

The survey consisted of two components: (1) a face-to-face interview, which elicited information on respondents’ characteristics, including their sociodemographic status and health-related behaviours and outcomes, followed by (2) a health examination comprising a series of anthropometric measurements such as measured height and weight, waist circumference, and body fat percent as well as other health measures. The face-to-face interview survey contained our main outcome measure of interest, the sex- and ethnicity-specific silhouette showcard of Pulvers and colleagues [21]. Specifically, the Pulvers silhouette showcards were based on the body image instrument specifically developed for populations of African descent (Fig. 1) and has shown to have a high inter-rater reliability (Cronbach α =0.95) and a high correlation with BMI amongst African American population [21]. This nine-image showcard presented sex-specific body sizes ranging from very thin (estimated BMI of 18 or less) to very obese (estimated BMI of 40 or higher). Specifically, perceived body size was measured based on participants’ response to the question “In this drawing, which figure best reflects how you think you look with regards to your weight?” and participants’ responses were recorded on a scale from one (for the thinnest silhouette) to nine (for the most obese silhouette). Participants were also asked about their current weight in kilograms, if known.

During the interview, information on average monthly earnings in Seychelles rupees (1 US$ ≈ 12 SRP in 2013) was collected. Income data was dichotomised using an 8000 SRP cut-off, representing average monthly earnings of our sample which also corresponded to average population earnings in 2013, according to the Seychelles National Bureau of Statistics [38]. Similarly, information on education was collected based on highest degree completed and dichotomised at the obligatory schooling cut-off, corresponding to the mean educational attainment of the sample. Education groups were those respondents with partial or completed obligatory schooling (zero to 11 years) and those with additional post-obligatory degrees from a polytechnic institute or university.

### Anthropometric measurement

We also analysed anthropometric data from the health examination component. Weight was measured with participants wearing light clothing and without shoes, using a calibrated medical electronic scale (Seca). Height was measured using a fixed stadiometer. Body fat percent was measured by the bioelectrical-impedance method (Omron Karada Scan BF504). Waist circumference was measured at the midpoint between the lower margin of the last palpable rib and the top of the iliac crest, while hip circumference was measured around the widest portion of the buttocks, with the tape parallel to the floor. BMI, waist to hip ratio, and waist to height ratio were calculated and used as body size and adiposity indicators [13].

### Statistical analysis

All analyses were performed separately for men and women using STATA SE 12 software (StataCorp, College Station, TX, USA). BMI was calculated as weight in kilograms divided by the square of height in meters. Body weight status was classified as underweight (BMI < 18.5 kg m^−2^), normal weight (BMI 18.5–24.9 kg m^−2^), overweight (BMI 25.0–29.9 kg m^−2^), or obese (BMI > 30.0 kg m^−2^) using the standard WHO categorization [39]. Box plots, fitted linear regression, and spearman correlations were used to describe associations between silhouette rankings and other body size and adiposity measures.

To evaluate the performance of silhouette rankings in detecting obesity, the BMI greater than or equal to 30.0 was used as the cut-off for obesity status [39]. We employed sex-specific receiver operating characteristic (ROC) analysis to assess silhouettes’ predictive validity compared to other body size and adiposity measures. The area under the curve (AUC) was used to compare the performance of silhouette rankings in predicting obesity with AUC of 1.0 indicating perfect discrimination, 0.9 excellent, 0.7 good, and 0.5 or less poor discrimination. Based on sensitivity and specificity estimates obtained from the ROC analyses, positive predictive values (PPV) and negative predictive values (NPV) were calculated to assess the diagnostic value of the silhouette rankings given the different prevalence of obesity in men and women.

To further assess the ability of the silhouettes rankings to detect the social patterning of body size and obesity, we also estimated two separate age-adjusted linear regression models, one using the silhouette rankings and the other with BMI as the outcome measure. The two binary socioeconomic predictors used in these models were respondents’ income and education level.

## Results

The descriptive sample statistics for men and women (Table 1) highlight significant sex differences in body sizes. The mean BMI for women was 29.3 compared to a mean BMI of 26.4 in men (*P* < 0.001). Similarly women had a higher total body fat percent of 41.1 % compared to 23.2 % in men (*P* < 0.001). The self-reported body size ranking based on the nine pictorial silhouettes also reflected similar sex differences in obesity with mean rankings of 4.0 and 5.2 in men and women, respectively (scale from one to nine). A similar proportion of men (65.0 %) and women (76.0 %) earn SR 8000 or less per month (approximately USD $640) and about 70.2 % of the population had obtained partial or complete compulsory school education.

**Table 1:** Sample characteristics by sex (Seychelles, 2013, *N* = 1240)

	Men (*N* = 531)	Women (*N* = 709)
	Mean (SD)	Mean (SD)
Age	46.3 (11.1)	45.2 (11.1)
Measured weight (kg)	79.4 (16.6)	76.3 (17.2)
Measured height (cm)	173.5 (6.6)	1613.3 (6.2)
Self-reported silhouettes (1/9)	4 (1.4)	5.2 (1.6)
Measured BMI (kg m^−2^)	26.4 (5.4)	29.3 (6.4)
Body Fat% (Bio-impedance)	23.2 (8.1)	41.1 (7.9)
Waist circumference (cm)	93 (13)	93.5 (13)
Hip circumference (cm)	102.3 (9.7)	108.7 (11.7)
Waist to height ratio	0.9 (0.1)	0.9 (0.1)
	Prevalence N (%)	Prevalence N (%)
BMI (kg m^−2^)		
Thinness (<18.5)	19 (3.6)	8 (1.1)
Normal (18.5–24.9)	204 (38.4)	172 (24.3)
Overweight (25−29.9)	196 (36.9)	241 (34.0)
Obese (30–60)	112 (21.1)	288 (40.6)
Income (in Seychells Rupees)		
Up to 8000 SR	345 (65.0)	539 (79.0)
More than 8000 SR	186 (35.0)	170 (24.0)
Education		
None or Obligatory	390 (73.4)	474 (66.9)
Polytechnic/University	141 (26.6)	253 (33.1)

Note: Values presented as mean, standard deviation (SD), and prevalence in %

When asked about their weight in kg, 31.5 % of respondents could not provide any information on their weight. The proportion of respondents who did not provide an answer to the weight question was higher in the low-income (52.1 %) and low-education (50.1 %) groups compared to the high-income (15.0 %) and high-education (13.9 %) segments of the population (*p* < 0.001). No significant difference in missing weight information was observed by sex or BMI status. Self-reported height was not included in survey. As a result, sociodemographic differences in self-reported height or BMI cannot be reported here. For those who provided an answer to the weight question, we obtained a discrepancy from −16.6 to +43 kg between measured and self-reported weight. The overall mean difference between measured and self-reported weight is −1.8 kg with no significant difference in under-reporting between women (−2.0, SD 5.35) and men (−1.5, SD 6.87).

**Fig. 2:**
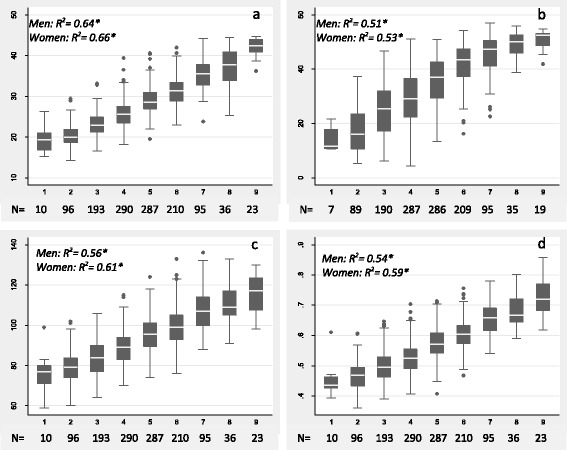
**a**-**d** Box plot relationships between self-reported silhouette ranking and selected adiposity measures (Seychelles, 2013, *N* = 1240). Note: measured BMI (**a**), fat percent (**b**), waist circumference (**c**), and waist to height ratio (**d**). Sex-specific linear regression R-squared values are presented with * when significance at <0.001. Number of participants selecting a given silhouette ranking is specified below each plot

For each silhouette category, the box plots in Fig. 2a-d indicate the conditional medians (horizontal lines in the boxes), the 25^th^ and 75^th^ percentiles (lower and upper hinges of the boxes), and lower and upper adjacent values (indicated by the ends of the whiskers in the charts) of the anthropometric measurements within each silhouette category. These box plots indicate that the medians and the 25^th^ and 75^th^ percentiles of the conditional distribution of subjects’ anthropometric measurements increased with increasing silhouette rankings for all four anthropometric adiposity measures. Fitting sex-specific linear regression models for each of the four anthropometric measures using the silhouette ranking as an explanatory variable showed a generally good fit overall (all R^2^ > 0.5). Moreover, the respective fits tend to be slightly better for women than men.

The Spearman correlation analysis of the relationship between the self-reported silhouette rankings and the other body size and adiposity measures yielded correlations ranging from 0.71–0.81 which are statistically significant in all four cases. The highest correlation coefficient was observed with BMI in men (0.8) and in women (0.81) and the lowest correlation was with body fat percent in men (0.71) and in women (0.73) (Table 2).

**Table 2:** Spearman correlation coefficients between self-reported silhouette ranking and selected adiposity measures (Seychelles, 2013, *N* = 1240)

	Men		Women	
	Correlation coefficient	*p*	Correlation coefficient	*p*
Body mass index (kg/m2)	0.80	<0.001	0.81	<0.001
Percentage body fat (%)	0.71	<0.001	0.73	<0.001
Waist circumference (cm)	075	<0.001	0.78	<0.001
Waist-to-height ratio	0.74	<0.001	0.77	<0.001

**Fig. 3:**
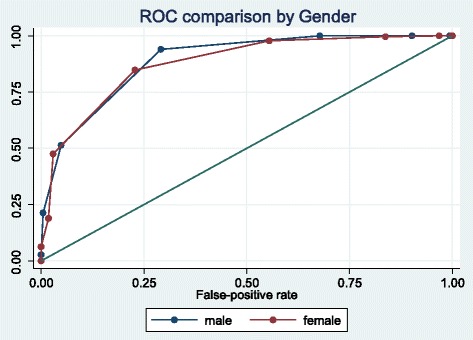
Performance of silhouettes for detection of obesity in men and women (Seychelles, 2013, *N*=1240). ROC curve to detect obesity (BMI of 30 or higher) using the 9-silhouette body size instrument in men and women

The accuracy of the silhouette show cards in detecting obesity (as per the standard cut-off BMI of 30) was analysed using sex-specific ROC analysis (Table 3). The silhouettes had an area under curve (AUC) of 0.91 in men and 0.89 in women. These AUC values were similar to those of waist circumference (AUC of 0.95 in men, and 0.94 in women), fat percent (AUC of 0.94 in men and women), and waist to height ratio (AUC of 0.95 in men and 0.94 in women) for detecting obesity (BMI > 30). The overall discriminatory power of silhouettes to detect obesity was thereby similar in both men and women (*P* < 0.001) (Fig. 3).

**Table 3:** Performance of self-reported silhouette ranking and other adiposity measures in detecting obesity by sex (Seychelles, 2013, *N* = 1240)

	Men (*N* = 531)			Women (*N* = 709)		
	AUC	SE	CI	AUC	SE	CI
Silhouettes	0.91	0.01	0.89–0.93	0.89	0.01	0.87–0.91
Waist circum.	0.95	0.01	0.94–0.97	0.94	0.01	0.92–0.95
Fat percent	0.94	0.01	0.92–0.96	0.94	0.01	0.92–0.96
Waist/Height	0.95	0.01	0.94	0.94	0.01	0.92–0.95

Note: Receiver Operator Characteristic (ROC) analysis resulting in Area Under the Curve (AUC), Standard Error (SE), and 95 % Confidence Interval (CI), for detection of obesity: BMI ≥ 30 kg m^−2^

Table 4 presents detailed estimates of sensitivity, specificity, and positive and negative predictive values for detecting obesity for each possible silhouette cut-off by sex. As the table shows, commonly proposed cut-offs for detecting obesity (cut-offs five or six) offers reasonable classifications of subjects as obese or not in terms of sensitivity, specificity, positive predictive value (PPV) and negative predictive value (NPV) for both men and women.

**Table 4:** Discriminatory ability of each self-reported silhouette ranking in detecting obesity by sex (Seychelles, 2013, *N* = 1240)

	Men				Women			
Cut off	Sensivity (%)	Specificity	PPV (%)	NPV (%)	Sensivity (%)	Specificity	PPV (%)	NPV (%)
(=1)	100	0.0	21.0	100	100	0.0	41.1	100
(=2)	100	1.9	21.3	100	100	0.5	41.1	100
(=3)	100	18.4	24.6	100	100	6.9	42.7	100
(=4)	100	45.1	32.6	100	99.3	25.7	48.1	98.5
(=5)	92.0	79.7	54.6	97.4	96.5	56.1	60.4	95.9
(=6)	46.4	97.1	81.2	87.2	80.9	84.1	77.9	86.4
(=7)	18.8	99.8	95.4	82.2	43.4	98.3	94.8	71.4
(=8)	4.5	100	100	79.7	17.4	99.1	92.7	63.3
(=9)	1.8	100	100	79.3	7.3	100	100	60.8

Note: Sensitivity, Specificity, Positive Predictive Value (PPV), and Negative Predictive Value (NPV) of each silhouette ranking in detecting obesity (BMI of 30 or higher) in men and women

Table 5 presents some further evidence on the ability of the self-reported silhouette rankings to detect the social patterning of body size. To this end, we compared sex-specific regression models for measured BMI on binary measures of income and education with corresponding models for the silhouette rankings on the same explanatory variables. While the magnitude of the coefficients are not directly comparable across regression models due to the different scales of the two outcome variables (measured BMI and silhouette rankings), we can still compare the regression coefficients’ signs, relative sizes, and statistical significance. As Table 5 shows, we obtained similar signs, relative sizes, and patterns of statistical significance for the income and education indicators in the regressions for measured BMI and the silhouette rankings for both men and women, highlighting the ability of the silhouette rankings to detect the socio-economic patterning of body sizes in our study population.

**Table 5:** Age-adjusted regression models for BMI and self-reported silhouette ranking on education and income (Seychelles, 2013, *N* = 1240)

	Men				Women			
	RC	SE	t test	*P* value	RC	SE	t test	*P* value
Measured BMI								
Income	2.44	0.51	4.82	0.00	−0.13	0.64	−0.21	0.84
Education	0.81	0.56	1.44	0.15	−2.46	0.06	−4.1	0.00
Self reported silhouette								
Income	0.62	0.13	4.74	0.00	−0.03	0.16	0.18	0.86
Education	0.22	0.14	1.50	0.14	−0.41	0.15	−2.7	0.01

Note: Comparison of silhouettes with measured BMI in detecting social pattering of obesity in men and women. (RC) Regression coefficients and Standard Error (SE)

## Discussion

Our study is the first validation study of Pulvers’ silhouette body size showcard as a simple self-reported survey measure for body size and adiposity in the adult population in the African region. Our findings reveal self-reported silhouette rankings to be comparable to other measured body size and adiposity indicators such as measured BMI, fat percent, waist circumference, and waist to height ratio. ROC analyses for both sexes further highlight the ability of self-reported silhouettes to serve as a good proxy for obesity. In addition, regression analyses show that silhouettes are also able to capture the social patterning of body size. The latter is particularly important in general-purpose social science survey settings, where the social patterning of body size is often of key interest. Our study thus highlights the usefulness of silhouette showcards for face-to-face general-purpose social science surveys when anthropometric measurements may not be feasible or cost-effective. While our study results are based on such face-to-face interviews, our findings also suggest the potential usefulness of silhouette showcards as a tool for assessing body size and obesity in mailed, online, or mobile device-based surveys, even though direct tests of the validity of silhouette showcards in the context of these alternative survey modes should still be conducted.

The rate of non-response of self-reported weight is rarely included in studies assessing the accuracy of self-reported weight [40, 41]. Such missing information and its associated factors can reveal important differences between subjects within a population. In our study, the differences between obtained and missing information on self-reported weight amongst various socioeconomic groups show that more than 50 % of people amongst lower education or income groups were not able to provide information on their weight status. This finding underlines the important challenges of collecting self-reported anthropometric data in low- and middle-income populations where obesity is a growing concern and where silhouette showcards can offer a cost-effective alternative to self-reported body size.

In our correlation analysis, we found self-reported silhouette rankings to be highly associated with other anthropometric measures of body size and adiposity such as BMI, waist circumference, waist to height ratio, and fat percent. Using Stunkard silhouettes, Bulik and colleagues [29] found similar Spearman correlations coefficients with BMI in Caucasians (0.73 for men and 0.81 for women) and Nagasaka and colleagues [27] also found correlations of 0.73 in men and 0.80 in women using a Japanese version of body shape silhouettes. The similarities of these different ethnically-specific silhouette showcards with measured BMI further suggest the relatively high degree of validity of showcards for measuring body size across different cultures and ethnicities.

Using BMI of 30 or greater as the cut-off for obesity, the self-reported silhouette rankings show a high accuracy for detecting obesity amongst African adults with AUC values of 0.91 and 0.89 in men and women, respectively. Pulvers and colleagues [36] found similar AUC in African American participants (AUC 0.88) and assigned the fifth silhouette to best correspond to the classification of overweight and obese individuals (BMI of 25 or higher) in African Americans. According to our ROC analysis, the expected performance in detecting obese subjects at the fifth silhouette reveals the sensitivity and specificity of 92.0 and 79.7 % in men and 96.5 and 56.1 % in women. However, when taking into account the prevalence of obesity, the sixth silhouettes resulted in better PPV and NPV proportions in both men (81.2 and 87.2) and women (77.9 and 86.4).

There are several limitations to our study which need to be considered when interpreting its findings or using it in actual survey design applications. First, while our tool was aimed at a population of African descent, about 10–20 % of our sample was likely from non-African descent (Caucasian, Indian, Chinese). Second, silhouette showcards have only a defined number of images, which tends to limit very thin and very obese individuals in their choice of appropriate images and may therefore not be suitable for those specific populations. Similarly, representing ordered silhouettes from thinnest to heaviest can also results in reporting bias, which suggests that jumbled-ordered cards may be preferred to reduce this potential bias. Third, while there are strong correlations between silhouette rankings and measured anthropometric indicators of body size, each silhouette does not necessarily have a direct correspondence with specific BMI values, which makes it challenging to choose BMI sub-classifications, such as measuring overweight status. More accurate BMI-based body size guides have been developed [42] and could be used in future validation studies to further assess the utility of silhouettes as a measure for BMI sub-classification of individuals in survey settings. Finally, our results may somewhat overstate the validity of silhouette showcards as a tool to measure body size given that our survey-based showcard instrument was administered during an in-person face-to-face interview that was followed by a health examination. Our setting may thus have prompted respondents to give more accurate answers on their body size than they would have done otherwise, even though our data still feature considerable misreporting and non-response with regard to respondents’ weight and height.

Despite the above limitations, we believe that our study provides new and valuable evidence on the performance of the silhouette showcards for measuring body size and its socioeconomic patterning by comparing silhouette-based outcome measurements with those of four classical anthropometric body size indicators: BMI, waist circumference, waist to hip ratio, and body fat percent.

Our study highlights the validity of silhouettes as a survey tool in a population in the African region. We found silhouettes to be a useful and inexpensive adiposity indicator in population studies when limited resources or other circumstances do not allow for direct anthropometric measurements. The good performance and ease of use of this tool makes it an attractive alternative to measured BMI for general-purpose social science surveys when direct measurement of weight and height is not cost-effective or feasible because of various circumstances, as well as for mail, online, or mobile device-based surveys.
